# Mining the chemical diversity of the hemp seed (*Cannabis sativa* L.) metabolome: discovery of a new molecular family widely distributed across hemp

**DOI:** 10.3389/fpls.2023.1114398

**Published:** 2023-08-10

**Authors:** Guillermo Federico Padilla-González, Abigail Rosselli, Nicholas J. Sadgrove, Max Cui, Monique S.J. Simmonds

**Affiliations:** ^1^ Enhanced Partnerships Department Royal Botanic Gardens, Kew, London, United Kingdom; ^2^ Department of Botany and Plant Biotechnology, University of Johannesburg (Auckland Park Campus), Auckland Park, Johannesburg, South Africa

**Keywords:** Cannabis, cinnamic acid amines, chemical diversity, metabolomics, HEMP, fatty acids

## Abstract

Hemp (*Cannabis sativa* L.) is a widely researched industrial crop with a variety of applications in the pharmaceutical, nutraceutical, food, cosmetic, textile, and materials industries. Although many of these applications are related to its chemical composition, the chemical diversity of the hemp metabolome has not been explored in detail and new metabolites with unknown properties are likely to be discovered. In the current study, we explored the chemical diversity of the hemp seed metabolome through an untargeted metabolomic study of 52 germplasm accessions to 1) identify new metabolites and 2) link the presence of biologically important molecules to specific accessions on which to focus on in future studies. Multivariate analysis of mass spectral data demonstrated large variability of the polar chemistry profile between accessions. Five main groups were annotated based on their similar metabolic fingerprints. The investigation also led to the discovery of a new compound and four structural analogues, belonging to a previously unknown chemical class in hemp seeds: cinnamic acid glycosyl sulphates. Although variability in the fatty acid profiles was not as marked as the polar components, some accessions had a higher yield of fatty acids, and variation in the ratio of linoleic acid to α-linolenic acid was also observed, with some varieties closer to 3:1 (reported as optimal for human nutrition). We found that that cinnamic acid amides and lignanamides, the main chemical classes of bioactive metabolites in hemp seed, were more concentrated in the Spanish accession Kongo Hanf (CAN58) and the French accession CAN37, while the Italian cultivar Eletta Campana (CAN48) demonstrated the greatest yield of fatty acids. Our results indicate that the high variability of bioactive and novel metabolites across the studied hemp seed accessions may influence claims associated with their commercialization and inform breeding programs in cultivar development.

## 1 Introduction

*Cannabis sativa* L., specifically the subspecies, varieties and cultivars known as hemp, are considered one of the most versatile herbaceous plants in agriculture, because of its commercial value to the pharmaceutical, food, nutraceutical, cosmetics, textile, papermaking, and construction industries (Schluttenhofer and Yuan, 2017; Farinon et al., 2020). In the context of its nutraceutical use, the focus has been on its seeds, which are rich in polyunsaturated fatty acids, proteins, minerals, and specialized metabolites with noteworthy implications for human and animal nutrition (Callaway, 2004; Russo and Reggiani, 2015; Galasso et al., 2016; Farinon et al., 2020). In this regard, hemp seeds are processed to make edible oil, cake flour and protein powder (Farinon et al., 2020; Leonard et al., 2021b).

Considering that some varieties of *C. sativa* express psychoactive cannabinoids that are occasionally detected in *Cannabis*-based products, a distinction between hemp and the recreational variety has been made (Schluttenhofer and Yuan, 2017). The European Industrial Hemp Association (EIHA) defines hemp as “the plant *Cannabis sativa* L., or any part of it, with a delta-9 tetrahydrocannabinol (THC) concentration up to 0.3% on a dry weight basis” to differentiate it from psychoactive *C. sativa* in which the THC concentration exceeds 0.3% and can be up to 20% (European Industrial Hemp Association, https://eiha.org/). Subsequently, several countries mainly from Europe, North America and Asia legalized the cultivation of low THC hemp cultivars (Leonard et al., 2021b). By 2018 more than 50,000 hectares were allocated to the cultivation of this crop in Europe, one of the main producers of industrial hemp. Within Europe, France is currently the largest producer, followed by Italy and the Netherlands (European Industrial Hemp Association, https://eiha.org/). Over the years, varieties of hemp were developed to select for specific traits and seeds from many of these varieties are currently stored in international germplasm collections and botanical gardens. There are currently 75 varieties registered in the EU Catalogue, all of them with a THC content below 0.3% and a diverse profile of other metabolites (European Industrial Hemp Association, https://eiha.org/).

Beyond the well-known cannabinoids, *C. sativa* contains a diverse metabolome of bioactive metabolites implicated in human health and nutrition. Hemp seeds are low in cannabinoids, and rich in phenylpropionamides (cinnamic acid amides and lignanamides) and unsaturated fatty acids, attracting nutraceutical and commercial interest (Crescente et al., 2018; Leonard et al., 2021a; Leonard et al., 2021b). The principal nutraceutical value of hemp seed oil is in its fatty acid composition, dominated by >90% of polyunsaturated fatty acids (Schluttenhofer and Yuan, 2017). It contains two dietary essential fatty acids, linoleic acid and α-linolenic acid in the ratio of 2.5–3:1. This is allegedly ideal for human nutrition and cardiovascular health (Leizer et al., 2000; Simopoulos, 2008; Galasso et al., 2016). However, the presence of high quantities of phenylpropionamides in hemp seeds have also been linked to some of their biological properties (Chen et al., 2012; Moccia et al., 2020; Leonard et al., 2021a). Hemp seeds accumulate the highest structural diversity of lignanamides among other lignanamides-producing taxa, with more than 80 different compounds (Leonard et al., 2021a).

Cinnamic acid amides and lignanamides display potent anti-inflammatory, antioxidant and anti-cancer activities in both *in-vitro* and *in-vivo* studies (Chen et al., 2012; Farinon et al., 2020; Moccia et al., 2020; Leonard et al., 2021a). Previous studies have promoted these hemp polyphenols as protective agents against human chronic diseases (Leonard et al., 2021a). For example, phenylpropionamides such as cannabisin B and N-*trans*-caffeoyltyramine are significantly stronger antioxidants than the standard soy isoflavones (Chen et al., 2012). Subsequent studies demonstrated that cannabisin B also enacts antiproliferative activity by inducing autophagic cell death in liver hepatocarcinoma (HepG2) cells (Chen et al., 2013). Other hemp lignanamides, such as cannabisin F, have demonstrated a potential neuro-protective effect by reducing mRNA levels of pro-inflammatory mediators, intracellular reactive oxygen species (ROS) and tumor necrosis factor α (TNF-α) in lipopolysaccharide-stimulated BV2 microglia cells (Wang et al., 2019).

Thus, the health and nutritional properties of hemp seeds are dependent upon their chemical composition that differs mainly according to the variety used. With such a high number of hemp varieties currently available in international germplasm collections, a comprehensive chemical assessment of these accessions is especially relevant, specifically to discover new metabolites and link the presence of biologically important molecules to certain accessions, ultimately to exploit the hemp germplasm by designing commercial cultivars with specific attributes. However, to date the phytochemical diversity of the hemp seed metabolome has not been explored in detail. In the present study, we focused on characterizing the chemical diversity of the hemp seed metabolome across cultivars/accessions using an untargeted metabolomics study of 52 hemp seed accessions.

## 2 Materials and methods

### 2.1 Plant material

A collection of 51 hemp seed accessions from fourteen countries were obtained from the Leibniz Institute of Plant Genetics and Crop Plant Research (IPK), Gatersleben, Germany (http://gbis.ipk-gatersleben.de/) (**Table 1**). These accessions, maintained *ex-situ* by the IPK, were provided as small quantities of seeds (< 1 g). An additional sample of hemp seeds (1 kg) (S1, **Table 1**) was acquired in a local supermarket for further isolation of targeted metabolites (see section 2.5), bringing the total number of samples to 52.

**Table 1:** Hemp seed accessions and cultivars included in the present study.

Code	Scientific name	Cultivar or Accession name	Country of origin	EU catalogue status
CAN16	*Cannabis sativa* L.	–	Slovakia	–
CAN17	*Cannabis sativa* L.	–	Hungary	–
CAN18	*Cannabis sativa* L. subsp. *sativa*	–	Germany	–
CAN19	*Cannabis sativa* L.	–	Italy	–
CAN20	*Cannabis sativa* L.	–	North Korea	–
CAN21	*Cannabis sativa* L. subsp. *sativa*	–	Romania	–
CAN22	*Cannabis sativa* L. subsp. *spontanea* Serebr.	–	Georgia	–
CAN23	*Cannabis sativa* L. subsp. *sativa*	–	North Korea	–
CAN24	*Cannabis sativa* L.	–	Italy	–
CAN26	*Cannabis sativa* L.	–	Turkey	–
CAN27	*Cannabis sativa* L.	–	unknown	–
CAN28	*Cannabis sativa* L.	Forose	unknown	–
CAN29	*Cannabis sativa* L. subsp. *sativa*	–	Romania	–
CAN30	*Cannabis sativa* L.	Bernburger	Germany	–
CAN31	*Cannabis sativa* L. subsp. *sativa*	Cunepa	Romania	–
CAN32	*Cannabis sativa* L. subsp. *sativa*	Conopla (ukr.)	Romania	–
CAN33	*Cannabis sativa* L. subs. *sativa*	Cinepa	Romania	–
CAN34	*Cannabis sativa* L. subs. *sativa*	Cinepa	Romania	–
CAN35	*Cannabis sativa* L. subs. *sativa*	Cinepa	Romania	–
CAN36	*Cannabis sativa* L. subsp. *sativa*	–	unknown	–
CAN37	*Cannabis sativa* L. subsp. *sativa*	–	France	–
CAN38	*Cannabis sativa* L. subsp. *sativa*	Cinepa	Romania	–
CAN39	*Cannabis sativa* L.	–	China	–
CAN40	*Cannabis sativa* L.	–	Italy	–
CAN42	*Cannabis sativa* L. subsp. *sativa*	–	Croatia	–
CAN43	*Cannabis sativa* L. subsp. *sativa*	Hohenthurmer Gleichzeitig Reifender	Germany	–
CAN44	*Cannabis sativa* L. subsp. *sativa*	Fibrimon	Germany	–
CAN45	*Cannabis sativa* L. subsp. *sativa*	Krasnodarskaya	Russia*	–
CAN46	*Cannabis sativa* L.	Fibrimon 56	France*	Deleted
CAN47	*Cannabis sativa* L. subsp. *sativa*	–	Turkey	–
CAN48	*Cannabis sativa* L.	Eletta campana	Italy	Registered
CAN50	*Cannabis sativa* L. subsp. *sativa*	Fibrimon	France*	–
CAN51	*Cannabis sativa* L. subsp. *sativa*	–	Argentina	–
CAN52	*Cannabis sativa* L. subsp. *sativa*	Fibridia	unknown	–
CAN53	*Cannabis sativa* L. subsp. *sativa*	Fibrimon	France*	–
CAN54	*Cannabis sativa* L. subsp. *sativa*	Fibrimon 21	France*	Deleted
CAN55	*Cannabis sativa* L. subsp. *sativa*	Havellander	Russia*	–
CAN56	*Cannabis sativa* L. subsp. *sativa*	Kompolti	Hungary*	Registered
CAN58	*Cannabis sativa* L. subsp. *sativa*	Kongo Hanf	Spain	–
CAN59	*Cannabis sativa* L. subsp. *sativa*	Turken P 494	unknown	–
CAN60	*Cannabis sativa* L. subsp. *sativa*	Schurig	Russia*	–
CAN61	*Cannabis sativa* L. subsp. *sativa*	Carmagnola in Selezione	Italy	Registered
CAN62	*Cannabis sativa* L. subsp. *sativa*	Bredemann P	Germany*	–
CAN63	*Cannabis sativa* L. subsp. *sativa*	Ramo	Germany	–
CAN64	*Cannabis sativa* L. subsp. *sativa*	–	Turkey	–
CAN65	*Cannabis sativa* L. subsp. *sativa*	Juso 14	Germany	Deleted
CAN66	*Cannabis sativa* L. subsp. *sativa*	Lovrin 110	Germany	Registered
CAN67	*Cannabis sativa* L. subsp. *sativa*	Fasamo	Germany	Deleted
CAN68	*Cannabis sativa* L. subsp. *sativa*	Kompolti	Germany	Registered
CAN69	*Cannabis sativa* L. subsp. *sativa*	Futura	Germany	Deleted
CAN70	*Cannabis sativa* L. subsp. *sativa*	Kompolti	Germany	Registered
S1	*Cannabis sativa* L.	–	unknown	–

* Country of origin not reported in the IPK database.

The 51 externally sourced samples derive from sixteen recognised cultivars, including five historic cultivars whose seeds have a “deleted” status (**Table 1**) in the EU database of registered plant varieties (https://ec.europa.eu/info/index_en). Samples that were attained in replicate include: three accessions of the cultivar “Fibrimon” (plus two related but deleted strains, Fibrimon 21 and 56), four of “Cinepa” and three of “Kompolti” (**Table 1**).

### 2.2 Extraction and LC-MS analyses

The extraction and metabolic profiling by LC-MS was based on the protocol reported by De Vos et al. (2007). Seeds from each of the hemp accessions were ground in liquid nitrogen with a pestle and mortar. Fifty milligrams of the resultant seed powder were then transferred to Eppendorf tubes and 2 mL of 80% MeOH (HPLC grade) were added as extraction solvent. Tubes were then placed in an ultrasonic bath for 15 min at 25°C using a frequency of 40 kHz. After extraction, samples were centrifuged at 19,975 × g for 10 min at room temperature, and the supernatant was transferred to an LC-MS vial. Samples were randomly analyzed by arbitrarily selecting vials to minimize statistical bias. Furthermore, 30 µL of each of the 52 samples were pooled into a LC-MS vial labelled as “Quality Control”. This sample was analyzed multiple times along the chromatographic sequence to check for reproducibility (De Vos et al., 2007).

The metabolic profiles of the hemp seed accessions were recorded on a Vanquish UHPLC system coupled to a 100 Hz photodiode array detector (PDA) and an Orbitrap Fusion Tri-hybrid high-resolution tandem mass spectrometer (Thermo Fisher Scientific, Waltham, MA, USA). Chromatographic separation of hemp seed extracts (5 μL) was performed on a Luna C18 column (150 mm × 3 mm i.d., 3 μm, Phenomenex, Torrance, CA, USA) using a linear mobile phase gradient of 0:90:10 to 90:0:10 [MeOH (A): water (C): acetonitrile +1% formic acid (D)] over 20 min at a flow rate of 400 μL min^−1^. Ultraviolet data were recorded between 210 nm and 550 nm.

Mass spectrometric detection was performed in the positive and negative ionization modes using the full scan and data dependent MS^2^ and MS^3^ acquisition modes. Total ion current (TIC) chromatograms were obtained over the range of 125 – 1800 *m/z* using a spray voltage of +3.5 and −2.5 kV for the positive and negative ionization modes, respectively. Four different scan events were recorded for each ionization mode as follows: (1) full scan, (2) MS^2^ of the most intense ion in *full scan*, (3) MS^3^ of the most intense ion in MS^2^, and (4) MS^3^ of the second most intense ion in MS^2^. Additional parameters for the MS included the following: full scan resolution set to 60,000 (full-width at half-maximum, FWHM), capillary temperature set to 350°C, ion transfer tube temperature set to 325°C, RF lens set to 50%, automatic gain control target set to 4.0 × 105 (*full scan*) or 1.0 × 104 (MS^2^ and MS^3^), intensity threshold set to 1.0 × 10^4^, collision-induced dissociation energy set to 35 eV, activation *Q* set to 0.25, and isolation window set to 4 *m*/*z*. Nitrogen was used as the drying, nebulizer and fragmentation gas.

### 2.3 LC-MS data processing and multivariate analysis

The LC-MS raw data was sliced into two sets according to the ionization mode (positive and negative) and transformed to mzXML format using the MSConvert package from the software ProteoWizard 3.0.9798 (Proteowizard Software Foundation, Palo Alto, CA, USA). Each data set was then processed by MZmine 2.53 (Pluskal et al., 2010) following the protocol previously described (Padilla-González et al., 2020a; Padilla-González et al., 2020b). After MZmine pre-processing, the results were exported as.csv and.mgf files which were then uploaded to the GNPS platform (https://gnps.ucsd.edu) for Feature Based Molecular Networking (FBMN) analysis (Wang et al., 2016; Nothias et al., 2020). The.csv file containing quantitative information related to ion abundances in each hemp seed sample was also submitted to multivariate statistical analysis by principal component analysis (PCA), hierarchical clustering analysis (HCA) and heatmaps in the software R 3.0.3 (R Foundation for Statistical Computing, Austria). Prior to the multivariate analyses the data matrix was centered and scaled by unit variance to avoid statistical bias influenced by differences between high and low abundant metabolites. LC-MS files, R scripts and detailed parameters used in MZmine (available as an MZmine batch file) are freely available in the MassIVE repository (study identifier MSV000090725).

### 2.4 Feature-based molecular networking and annotation tools

Feature-based molecular networking (FBMN) was performed following the workflow by (Nothias et al., 2020) on the GNPS platform (https://gnps.ucsd.edu). For this analysis, the data were first filtered by removing all MS/MS fragment ions within a window of +/−17 Da of the precursor ion value and by choosing only the top 6 fragment ions in the +/− 50 Da window throughout the spectrum. The precursor and fragment ion mass tolerance were set to 0.05 Da and 0.99 Da respectively, in accordance with the MS settings used. A cosine score above 0.65 and more than six matched peaks were considered for creating the molecular network. Furthermore, edges between two nodes were kept in the network only if each of the nodes appeared in each other’s respective top 10 most similar nodes. Experimental MS^2^ spectra were then searched against the GNPS spectral libraries (Wang et al., 2016) using the same filters as for the input data. To enhance chemical structural information within the molecular network and provide a more comprehensive overview of the hemp seed chemical space, information from *in silico* structure annotations from the GNPS library search were incorporated into the network using the GNPS MolNetEnhancer and MS2LDA workflows (Wandy et al., 2018; Ernst et al., 2019). MolNetEnhancer and MS2LDA were performed using the default values reported in GNPS, except for bin width in MS2LDA, which was set at 0.1. Chemical class annotations were performed using the ClassyFire chemical ontology (Feunang et al., 2016). Final molecular networks including quantitative data, substructure information and chemical classifications were then visualized using the software Cytoscape (Ono et al., 2014).

Lastly, to confirm and expand the spectral library annotation made by molecular networking and its *in-silico* match against the GNPS spectral libraries, accurate mass values, MS^2^ and MS^3^ data of all the annotated metabolites were manually inspected and compared with information available in the literature to achieve different confidence values following the Metabolomics Standards Initiative (Sumner et al., 2007).

### 2.5 Isolation of new metabolites

One kilogram of seeds was acquired in a local supermarket and submitted to an MS-guided isolation protocol to target the isolation of a potentially new metabolite detected by LC-MS, following the same steps as done previously (Padilla-González et al., 2017). Briefly, the seeds were grinded into a fine power and extracted with 80% water in methanol for 24 h, with two repeat extractions of the same material, to exhaust the polar metabolome from the seed flour. Extracts were combined and then evaporated on a centrifugal evaporator (Genevac EZ-2, SP Industries, Warminster, PA, USA) to remove the organic solvent fraction and then lyophilized to remove water and yield a dry crude extract residue. Ten grams of the extract were submitted to Sephadex LH-20 column chromatography (120 g, 400 × 40 mm i.d.) employing mixtures of 200 mL of water–methanol (100:0, 98:2, 96:4, 94:6, and 50:50) that were combined to afford ten fractions (Fr1–Fr10) after LC-UV-MS monitoring for similarities. Fr2 was further purified by semipreparative HPLC (Waters e2695, Waters, Milford, MA, USA) using a Luna C18 column (250 × 10 mm i.d., 10 µm, Phenomenex) and a mobile phase of acetonitrile and water both acidified with 0.1% of formic acid. Chromatographic separation was performed using a linear gradient of 5 – 20% acetonitrile over 30 min, to yield 4 mg of ferulic acid 4-*O*-glucosyl-6’-*O*-sulphate. The structure of the isolated metabolite was determined using uni- and bidimensional NMR experiments (Bruker ARX 400, Billerica, MA, USA, MeOD) and by high-resolution MS (Orbitrap Fusion, Thermo Scientific) and low-resolution tandem mass spectrometry (Ion Trap Velos Pro, Thermo Scientific).

### 2.6 Extraction and derivatization of fatty acids

Powdered seeds (100 mg) from each hemp accession were extracted with 2 mL of *n*-hexane in an ultrasonic bath for 15 min at 25°C using a frequency of 40 kHz. After extraction, samples were evaporated under vacuum to obtain an oily residue corresponding to hemp seed fixed oil. Five microliters of the oil were added to a 2 mL glass vial containing 500 µL of boron trifluoride in methanol (20%). The vial was filled with nitrogen, tightly sealed and gently mixed. The sample vial was subsequently heated at 100 °C for 15 min in a heating block. After cooling to room temperature, 500 µL of water and 750 µL of pentane were added to the vial and vigorously shaken. The organic phase (upper) was then transferred to another vial and evaporated under nitrogen. This partition was repeated for a second time and the organic phases were mixed and evaporated under nitrogen. The concentrated sample containing fatty acids methyl esters was re-dissolved in 200 µL of *n*-hexane and placed in a glass vial with insert for GC-MS analysis.

### 2.7 GC-MS analyses of fatty acids methyl esters

GC-MS analyses of fatty acids methyl esters were performed in an Agilent 7890A GC chromatograph coupled to a single quadrupole MS analyser (Agilent 5975C). Chromatographic separation was performed on a ZB-WAX column (30 m x 0.25 mm i.d. x 0.25 µm, Phenomenex), using helium as carried gas at a flow rate of 1 mL per minute. The temperature program was configured from 70 – 250 °C at a linear increment of 3 °C per minute. The injection temperature was set to 220 °C and the source temperature to 180 °C. 70 eV was used as ionization energy and the mass spectrometer was configured to record data from 38 to 650 *m/z.* Compounds were identified by comparing their retention indices (calculated against a series of *n*-alkanes analysed under the same experimental conditions) and by comparing their mass spectra with the NIST library.

The percentage composition of the fatty acid methyl esters detected in each hemp cultivar/accession was semi-quantitatively obtained by manual integration of GC-MS data. This information was then centered and scaled by unit variance and submitted to statistical analyses in the software R.

## 3 Results

### 3.1 Metabolic profiling by LC-MS/MS

#### 3.1.1 Exploratory methods and mining of the hemp seed metabolome

**Figure 1:**
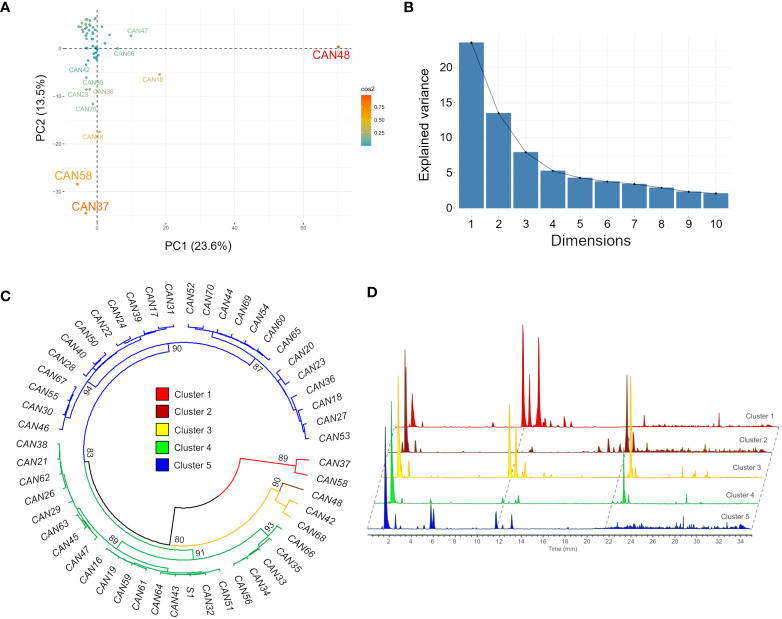
Clustering of the fifty-two hemp seed accessions based on the similarities of their metabolic fingerprints detected by LC-MS in positive ion mode. **(A)** PCA scores plot showing accessions CAN48, CAN58 and CAN37 as outliers; **(B)** Scree plot and the percentage of variance explained by the first 10 components; **(C)** HCAbp showing the metabolic clustering of the 52 hemp seed accessions in five main clusters highlighted in different colours and **(D)** Overlaid LC-MS chromatograms of one representative sample from each of the five groups.

Metabolic profiling by LC-MS and concomitant data processing by MZmine 2.53 of 52 hemp seed accessions detected 437 and 242 mass features in the positive and negative ionization modes, respectively. A principal component analysis (PCA) of the positive mode dataset explained 37.1% of the total variance in the first two components and grouped the seed extracts according to the similarities of their metabolic fingerprints (**Figures 1A, B**). This analysis revealed that while most accessions have similar metabolic profiles, the Italian cultivar Eletta Campana (CAN48), the Spanish accession Kongo Hanf (CAN58) and the French accession CAN37 (**Table 1**) are clear chemical outliers (**Figure 1A**).

A hierarchical clustering analysis with bootstrap resampling (HCAbp) grouped the 52 hemp seed accessions into five main clusters with support values >80% (clusters 1-5, **Figure 1C**). This analysis further confirmed that accessions CAN37 and CAN58 form an independent group chemically different from the remaining hemp seed accessions (cluster 1, **Figure 1C**), while the Italian cultivar Eletta Campana (CAN48) is closely related in chemical composition to the Croatian accession (CAN42) and the German cultivar Kompolti (CAN68) (**Figure 1C**). Similar results were obtained in the PCA and HCAbp of the negative mode dataset with accessions CAN37 and CAN58 and cultivars CAN48 and CAN68 as clear outliers based on their metabolic fingerprints (**Figure S1**). It is noteworthy that the chemical differences between the samples in groups 1 – 5 are visibly distinguishable by interrogation of the base peak chromatograms. To exemplify this, a representative sample from each group is provided in **Figure 1D**.

Close inspection of the five HCA-based groups reveals that in some cases a correlation of chemical composition with the hemp seed cultivar/accession occurs. For example, the five Fibrimon cultivars included in the present study clustered in group 5, while all Cinepa accessions clustered in group 4. This pattern, however, was not consistent across all hemp cultivars, since the three investigated Kompolti samples were grouped in different clusters (**Figure 1C**). The correlation between chemical compositions and country of origin was also not evident. The eleven German accessions included in the present study were distributed across three different clusters, as were the four Italian accessions (**Figure 1C**).

**Figure 2:**
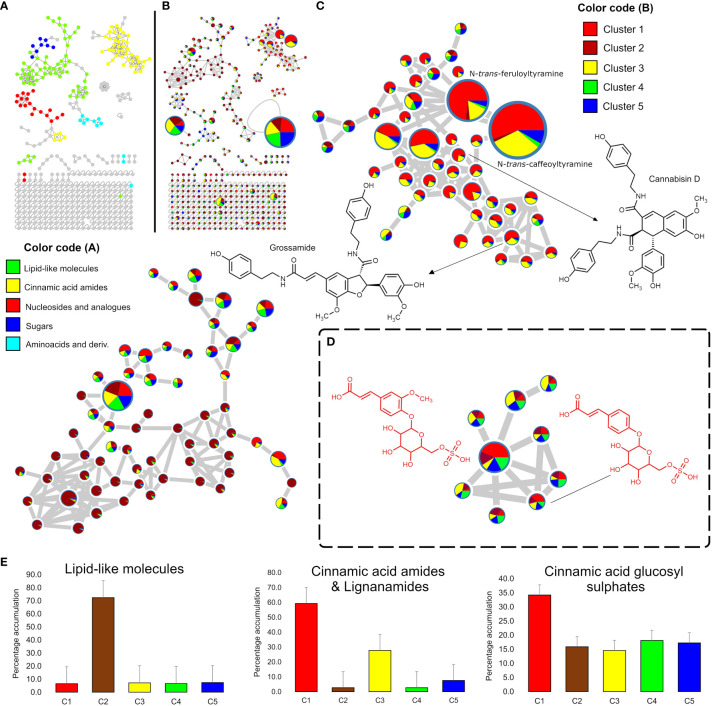
Mining of the hemp seed metabolome by Feature Based Molecular Networking (FBMN) of LC-MS data. **(A)** FBMN showing the chemical class assigned to each network through the MolNetEnhancer analysis of the positive ion mode LC-MS data; **(B)** FBMN showing pie charts representing the proportional accumulation of each node (representing *m/z* values) in the five groups identified by HCAbp (**Figure 1C**). The node size is proportional to the ion intensity. **(C)** Amplified nodes of cinnamic acid amides and lignanamides and lipid-like molecules detected in the positive ion mode dataset. **(D)** Amplified nodes of a previously unreported chemical class in hemp “Cinnamic acid glycosyl sulphates” detected only in the negative ion mode. **(E)** Bar charts summarizing the percentage accumulation of lipid-like molecules, cinnamic acid amides and lignanamides and cinnamic acid glycosyl sulphates in the five HCAbp clusters.

A feature-based molecular networking analysis (FBMN) with *in silico* annotation tools of the positive mode LC-MS dataset revealed a clustering tendency by chemical class, where cinnamic acid amides (including lignanamides) and lipid-like molecules clustered the higher number of nodes (**Figure 2A**). Cinnamic acid amides and lipid-like molecules were consistently the most structurally diverse chemical classes in hemp seeds. Other classes detected include nucleosides and analogues, sugars and amino acids with derivatives (**Figure 2A**). The FBMN analysis, along with database searches and manual inspection of MS data, facilitated the identification of the majority of previously reported cinnamic acid amides in hemp (**Table 2**) although some nodes from the same chemical class were not identified. This analysis also revealed the accumulation patterns of cinnamic acid amides in the different groups of hemp seed accessions maintained *ex-situ* in the IPK germplasm collection (**Figure 2B**). As observed in **Figure 2C**, while most of the nodes representing cinnamic acid amides are accumulated in all hemp seed accessions, the Spanish accession Kongo Hanf (CAN58) and the French accession CAN37 in cluster 1 seem to accumulate the highest levels of this chemical class with close to 60% of the total ion intensities, while intermediate levels (*ca*. 30%) are found in the accessions in cluster 3 (CAN42 and CAN68) (**Figure 2E**). The remaining hemp seed accessions collectively account for only *ca*. 13% of the total ion counts for this chemical class (**Figure 2E**). Regarding the lipid-like molecules, the Italian cultivar Eletta Campana (CAN48) showed the highest accumulation of this chemical class with 72% of the total ion counts, while the remaining 51 accessions together represent only 28% of the total ion counts (**Figure 2E**).

**Table 2:** Compounds annotated in a collection of 52 hemp seed accessions.

#	Rt	Class	Annotation	Molecular formula	Positive ionization	Positive MS^2^ ions (intensity)	Negative ionization	Negative MS^2^ ions (intensity)	Error (ppm)	Reference	MSI^a^
1	1.65	Sugars	Sucrose	C_12_H_22_O_11_	[M+NH_4_]^+^ 360.15067	325 (100), 343 (46), 289 (17), 307 (11), 259 (7), 198 (7), 145 (5)	[M - H]^-^ 341.10840	179 (100), 161 (21), 143 (16), 113 (14), 101 (7), 131 (6)	0.04	Current study	1
2	2.50	Organic acids	Citric acid	C_6_H_8_O_7_	–	–	[M - H]^-^ 191.01965	111 (100), 172 (24)	2.46	Current study	1
3	4.02	Phenolic acids	Protocatechuic acid hexoside	C_13_H_16_O_9_	–	–	[M - H]^-^ 315.07214	152 (100), 271 (21), 165 (13), 253 (12), 108 (12), 225 (9), 243 (7), 299 (5)	1.69	Kew database	2
4	5.19	Phenylpropionamides	Coumaroyl-amino butanol glucopyranoside	C_19_H_27_O_8_N	[M + H]^+^ 398.18103	147 (100), 309 (65), 291 (14), 236 (14)	–	–	1.17	(Moccia et al., 2020)	2
5	6.84	Glucosyl sulphates	Ferulic acid 4-*O*-glucosyl-6’-*O*-sulphate (Cannabigail)	C_16_H_20_O_12_S	–	–	[M - H]^-^ 435.06061	241 (100), 223 (5), 193 (5)	0.78	Current study	1
6	5.88	Glucosyl sulphates	Caffeic acid glucosyl sulphate	C_15_H_18_O_12_S	–	–	[M - H]^-^ 421.04361	241 (100), 179 (10)	1.10	Current study	3
7	6.23	Glucosyl sulphates	Cinnamic acid glucosyl sulphate (Cuinnabis)	C_15_H_18_O_11_S	–	–	[M - H]^-^ 405.04938	241 (100), 325 (5), 223 (4), 139 (4), 163 (3)	0.81	Current study	3
8	7.85	Glucosyl sulphates	Dimethoxy cinnamic acid glucosyl sulphate	C_17_H_22_O_13_S	–	–	[M - H]^-^ 465.07037	241 (100), 223 (5)	0.18	Current study	3
9	8.44	Glucosyl sulphates	Ferulic acid glucosyl sulphate isomer	C_16_H_20_O_12_S	–	–	[M - H]^-^ 435.05972	241 (100), 193 (5)	1.26	Current study	3
10	10.31	Cinnamic acid amides	N-*trans*-feruloyltyramine glycoside	C_24_H_29_NO_9_	[M + H]^+^ 476.19138	314 (100), 177 (8), 145 (2)	[M - H]^-^ 474.17648	312 (100)	1.42	(Leonard et al., 2021b)	2
11	11.69	Cinnamic acid amides	N-*trans*-caffeoyltyramine	C_17_H_17_NO_4_	[M + H]^+^ 300.12317	163 (100), 121 (10), 138 (9)	[M - H]^-^ 298.10846	135 (100), 178 (46), 161 (38), 256 (12), 107 (6)	1.38	(Leonard et al., 2021b)	2
12	13.13	Cinnamic acid amides	N-*trans*-feruloyltyramine	C_18_H_19_NO_4_	[M + H]^+^ 314.13882	177 (100), 145 (15)	[M - H]^-^ 312.12427	297 (100), 178 (92), 135 (44), 148 (18), 270 (15), 176 (12), 191 (8), 312 (6), 253 (5),	1.32	(Leonard et al., 2021b)	2
13	12.90	Cinnamic acid amides	N-*trans*-coumaroyltyramine	C_17_H_17_NO_3_	[M + H]^+^ 284.12814	147 (100)	[M - H]^-^ 282.11328	119 (100), 145 (80), 162 (50), 134 (21), 240 (12)	1.85	(Leonard et al., 2021b)	2
14	14.89	Cinnamic acid amides	tri-*p*-coumaroylspermidine	C_34_H_37_N_3_O_6_	[M + H]^+^ 584.27612	420 (100), 438 (80)	[M - H]^-^ 582.26062	462 (100), 342 (12), 436 (9)	0.09	(Nigro et al., 2020)	2
15	8.95	Cinnamic acid amides	N-*trans*-caffeoyloctopamine	C_17_H_17_NO_5_	[M + H]^+^ 316.11804	298 (100), 255 (10), 168 (8)	[M - H]^-^ 314.10349	296 (100), 202 (10)	1.44	(Leonard et al., 2021b)	2
16	10.11	Lignanamides	N- caffeoyltyramine/N- caffeoyloctopamine dimer	C_34_H_32_N_2_O_9_	[M + H]^+^ 613.21814	595 (100), 460 (12), 476 (4)	[M - H]^-^ 611.20239	593 (100), 430 (26), 501 (10), 320 (10), 448 (7)	0.75	(Nigro et al., 2020)	3
17	12.31	Lignanamides	Cannabisin B	C_34_H_32_N_2_O_8_	[M + H]^+^ 597.22308	460 (100), 432 (4)	[M - H]^-^ 595.20789	–	1.03	(Leonard et al., 2021b)	2
18	12.69	Lignanamides	Cannabisin B isomer	C_34_H_32_N_2_O_8_	[M + H]^+^ 597.22290	460 (100), 432 (20), 350 (9)	[M - H]^-^ 595.20746	485 (100), 432 (15) 322 (15), 456 (4)	1.33	(Leonard et al., 2021b)	3
19	12.91	Lignanamides	Cannabisin B isomer	C_34_H_32_N_2_O_8_	[M + H]^+^ 597.22369	460 (100), 297 (20), 434 (7), 418 (5), 279 (4)	[M - H]^-^ 595.20728	485 (100), 432 (92) 322 (28), 458 (14), 416 (14), 269 (12)	0.008	(Leonard et al., 2021b)	3
20	13.95	Lignanamides	3’3-demethylgrossamide	C_34_H_32_N_2_O_8_	[M + H]^+^ 597.22357	434 (100), 297 (40), 460 (32), 323(13) 271 (8), 279 (6)	[M - H]^-^ 595.20837	432 (100), 458 (19), 269 (12), 338 (5)	0.21	(Leonard et al., 2021b)	2
21	14.82	Lignanamides	Cannabisin M	C_34_H_32_N_2_O_8_	[M + H]^+^ 597.22345	434 (100), 297 (78), 300 (44), 460 (39), 187 (8)	[M - H]^-^ 595.20721	298 (100), 432 (5)	0.41	(Leonard et al., 2021b)	2
22	14.96	Lignanamides	Cannabisin Q	C_34_H_32_N_2_O_8_	[M + H]^+^ 597.22327	434 (100), 297 (95), 460 (55), 300 (30) 279 (4), 187 (4)	[M - H]^-^ 595.20715	298 (100), 432 (5)	0.71	(Leonard et al., 2021b)	2
23	12.24	Lignanamides	Cannabisin A	C_34_H_30_N_2_O_8_	[M + H]^+^ 595.20770	458 (100), 350 (2)	[M - H]^-^ 593.19159	456 (100), 430 (38), 293 (4)	1.35	(Nigro et al., 2020)	2
24	12.04	Lignanamides	Rac-sativamide A	C_33_H_30_N_2_O_8_	[M + H]^+^ 583.20758	565 (100), 446 (87), 418 (33), 255 (8)	[M - H]^-^ 581.19086	400 (100), 513 (8), 535 (7), 418 (6), 443 (5)	0.76	(Leonard et al., 2021b)	2
25	12.69	Lignanamides	Cannabisin H	C_28_H_31_NO_8_	[M + H]^+^ 510.21204	492 (100), 312 (42), 462 (35), 355 (37), 177 (8), 201 (7), 474 (6), 338 (5)	[M - H]^-^ 508.19772	312 (100), 460 (68), 490 (8), 366 (6)	1.48	(Leonard et al., 2021b)	2
26	14.43	Lignanamides	Grossamide K	C_28_H_29_NO_7_	[M + H]^+^ 492.20178	462 (100), 325 (37), 337 (14), 355 (8)	[M - H]^-^ 490.18640	472 (100), 460 (13)	0.90	(Leonard et al., 2021b)	2
27	13.04	Lignanamides	Cannabisin C	C_35_H_34_N_2_O_8_	[M + H]^+^ 611.23846	474 (100), 446 (4)	[M - H]^-^ 609.22308	446 (100), 283 (20), 499 (8)	1.45	(Leonard et al., 2021b)	2
28	13.53	Lignanamides	Cannabisin C isomer	C_35_H_34_N_2_O_8_	[M + H]^+^ 611.23859	474 (100), 311 (32), 448 (12), 432 (6)	[M - H]^-^ 609.22260	446 (100), 283 (12), 485 (5), 322 (5)	1.23	(Leonard et al., 2021b)	3
29	14.56	Lignanamides	Cannabisin N or isomer	C_35_H_34_N_2_O_8_	[M + H]^+^ 611.24005	448 (100), 311 (34), 474 (29), 337 (19), 285 (8), 293 (5), 355 (4)	[M - H]^-^ 609.22333	446 (100), 283 (15), 472 (5)	1.45	(Leonard et al., 2021b)	3
30	15.48	Lignanamides	Cannabisin N or isomer	C_35_H_34_N_2_O_8_	[M + H]^+^ 611.23877	448 (100), 311 (60), 474 (42), 314 (32), 187 (10), 298 (7)	[M - H]^-^ 609.22229	312 (100), 417 (76), 296 (69), 446 (32), 501 (16), 581 (16), 591 (10)	0.94	(Leonard et al., 2021b)	3
31	15.75	Lignanamides	Cannabisin N or isomer	C_35_H_34_N_2_O_8_	[M + H]^+^ 611.23822	311 (100), 448 (78), 474 (56), 324 (6), 293 (6)	[M - H]^-^ 609.22235	312 (100), 296 (62), 446 (38), 417 (12), 473 (10)	1.84	(Leonard et al., 2021b)	3
32	13.71	Lignanamides	Cannabisin D	C_36_H_36_N_2_O_8_	[M + H]^+^ 625.25446	488 (100), 460 (6)	[M - H]^-^ 623.23877	460 (100), 283 (7), 446 (7)	0.85	(Leonard et al., 2021b)	2
33	14.21	Lignanamides	Cannabisin G	C_36_H_36_N_2_O_8_	[M + H]^+^ 625.25409	488 (100), 460 (6)	[M - H]^-^ 623.23907	460 (100), 446 (20), 336 (12), 499 (9)	1.44	(Leonard et al., 2021b)	2
34	15.46	Lignanamides	Cannabisin F	C_36_H_36_N_2_O_8_	[M + H]^+^ 625.25397	462 (100), 325 (41), 488 (10), 351 (9), 307 (5), 299 (5)	[M - H]^-^ 623.23853	460 (100), 297 (20), 486 (12)	1.64	(Leonard et al., 2021b)	2
35	16.08	Lignanamides	Grossamide	C_36_H_36_N_2_O_8_	[M + H]^+^ 625.25433	488 (100), 462 (48), 351 (13), 325 (7)	[M - H]^-^ 623.23871	592 (100), 433 (37), 551 (28), 471 (24), 460 (22), 503 (12), 515 (8)	1.06	(Leonard et al., 2021b)	2
36	14.35	Lignanamides	Cannabisin E	C_36_H_38_N_2_O_9_	[M + H]^+^ 643.26483	462 (100), 625 (19), 338 (7), 488 (5)	[M - H]^-^ 641.24988	489(100), 623 (82), 431 (12), 312 (11), 460 (9), 328 (9), 517 (6)	1.12	(Leonard et al., 2021b)	2
37	14.64	Lignanamides	*cis*-Cannabisin E	C_36_H_38_N_2_O_9_	[M + H]^+^ 643.26495	462 (100), 625 (24), 338 (8), 478 (8), 450 (5)	[M - H]^-^ 641.24908	489 (100), 623 (75), 431 (11), 312 (11), 460 (10), 517 (6),	0.94	(Leonard et al., 2021b)	2
38	16.25	Lignanamides	Cannabisin O	C_54_H_53_N_3_O_12_	[M + H]^+^ 936.36957	799 (100), 771 (5)	[M - H]^-^ 934.35425	902 (100), 771 (80), 765 (54), 739 (38), 797 (25), 826 (10), 641 (8)	1.23	(Leonard et al., 2021b)	2
39	21.82	Cannabinoids	Cannabidiol or Tetrahydrocannabinol isomer	C_21_H_30_O_2_	[M + H]^+^ 315.23193	not fragmented	–	–	1.51	(Citti et al., 2019)	3
40	23.35	Cannabinoids	Tetrahydrocannabinol	C_21_H_30_O_2_	[M + H]^+^ 315.23187	297 (28), 259 (100), 235 (40), 233 (42), 221 (32), 207 (22), 193 (82), 181 (32), 135 (32)	–	–	1.70	(Citti et al., 2019)	3
41	22.16	Cannabinoids	Tetrahydrocannabinolic acid	C_22_H_30_O_4_	[M + H]^+^ 359.22186	[MS^2^] 341 (100), [MS^3^] 299 (10), 285 (48), 261 (72), 247 (8), 233 (30), 219 (100), 207 (12), 135 (4)	–	–	1.04	(Citti et al., 2019)	3
42	20.13	Lipids	Unknown fatty acid	C_18_H_32_O_4_	[M + H]^+^ 313.23755	295 (100), 277 (54), 227 (16), 209 (8), 259 (4)	–	–	1.07	–	4
43	21.78	Lipids	Unknown fatty acid	C_18_H_30_O_3_	[M + H]^+^ 295.22678	277 (100), 257 (7), 237 (3), 221 (3)	–	–	1.81	–	4
44	22.35	Lipids	Unknown fatty acid	C_18_H_32_O_3_	[M + H]^+^ 297.24258	279 (100), 261 (6), 243 (3), 163 (3)	–	–	1.29	–	4

a Confidence level of identification according to the Metabolomics standards initiative (1: Rt, HRMS and MS^2^ match with a pure standard; 2: HRMS and MS^2^ match with literature information; 3: tentative identity suggested by interpretation of MS^2^ spectra and literature information; unknown compounds).

FBMN of the negative ionization mode revealed similar patterns to the positive mode dataset with cinnamic acid amides clustering the higher number of nodes (**Figure S2**). However, several unidentified compounds were detected in this mode grouping into a previously unreported “molecular family” in hemp seeds (**Figure 2D**). Manual dereplication of the nodes belonging to this chemical class and further isolation and structure elucidation of targeted metabolites (see section 3.1.3) allowed the identification of this molecular family as “cinnamic acid glycosyl sulphates”. This unusual chemical class includes several structurally related metabolites, characterized by the presence of a cinnamic acid moiety (or a related derivative) *para*-*O*-linked to a glycosyl sulphate unit (**Figure 2D**). Interestingly, all hemp seed accessions showed consistently similar amounts of glycosyl sulphates with 15% to 20% of the total ion counts, except for the accessions in cluster 1 (CAN37 and CAN58) which showed a percentage accumulation of *ca*. 34% (**Figure 2E**).

#### 3.1.2 Metabolic differences among hemp seed accessions

**Figure 3:**
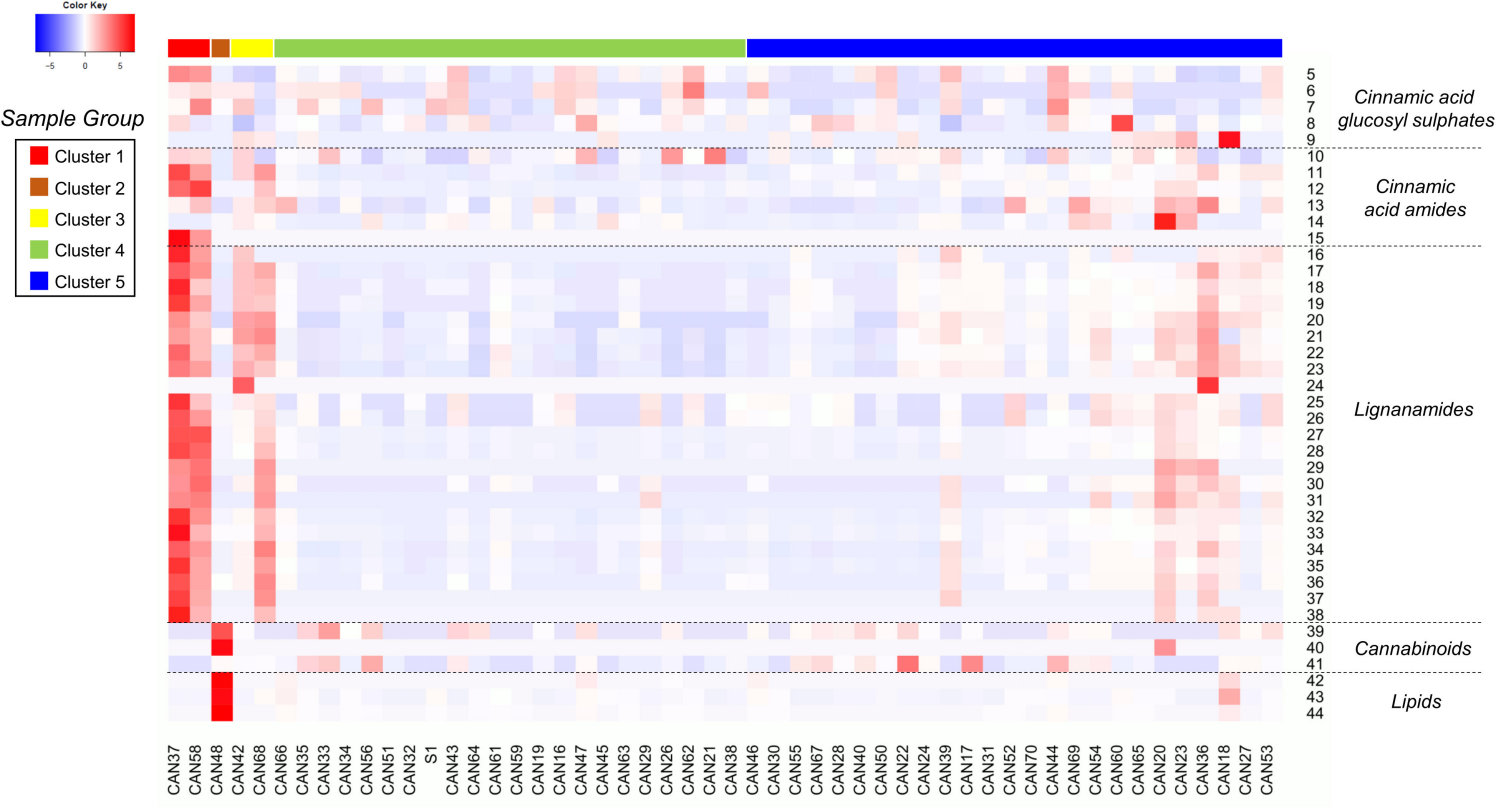
Heatmap showing the relative accumulation of the annotated metabolites in the 52 hemp seed accessions analysed by LC-MS. For compounds identities refer to **Table 2**.

Although the current study provides a snapshot of the metabolic composition of different hemp seed accessions obtained from plants grown in different conditions and geographic locations, we aimed to compare their metabolic profiles to link the presence of biologically important molecules to certain accessions in order to inform future studies. Therefore, to have a deeper understanding of semiquantitative differences among the annotated metabolites in each hemp seed accession, a heatmap analysis based on manually extracted peak intensities was performed (**Figure 3**). The annotation of these compounds was performed by HRMS and MS^2^ spectral matching with literature information and with spectra available in the GNPS database and in our *in*-house library of MS^2^ data. After *in-*silico spectral matching, manual inspection of annotated peaks was performed to confirm and expand the identifications. This combined analysis allowed the annotation of 44 metabolites belonging to five main chemical classes: Cinnamic acid glycosyl sulphates, cinnamic acid amides and their oxidative coupling products, lignanamides, cannabinoids and lipids (**Table 2**). These compounds were annotated with different levels of confidence according to the metabolomics standards initiative (Sumner et al., 2007).

##### 3.1.2.1 Cinnamic acid glycosyl sulphates

Detailed analyses of the metabolites assigned as cinnamic acid glycosyl sulphates allowed the annotation of four new compounds (5, 6, 8 and 9) and one known metabolite (compound 7). The assigned structures of compounds 6 – 9 are based on the absolute chemical structure of compound 5, ferulic acid 4-*O*-glucosyl-6’-*O*-sulphate (cannabigail), which was purified and assigned by 1D and 2D NMR interpretation in MeOD, supported by HRMS and MS^2^ data (see section 3.2. for details about its structural elucidation). All the compounds in this chemical class (compounds 5 – 9, **Table 2**) showed similarities in their MS^2^ spectra, characterized by the presence of a base peak ion at 241 *m/z* [M−H]^−^ (**Table 2**), suggesting close similarities in their chemical structures. However, detailed analyses of MS data revealed that the phenylpropanoid moiety in these compounds differed, being ferulic acid in compounds 5 and 9, caffeic acid in 6, cinnamic acid in 7 (cuinnabis) and dimethoxycinnamic acid in 8. Analysis of accumulation patterns of this chemical class in the 52 hemp seed accessions showed that the accumulation of cinnamic acid glycosyl sulphates is rather homogeneous across all hemp seed accessions, although the Spanish Kongo Hanf (CAN58) and the French accession CAN37 (cluster 1) accumulate an overall higher proportion of these metabolites. Interestingly, an inverse tendency in the accumulation of compound 5 and its isomer, compound 9, was observed across accessions CAN37 and CAN18, while compounds 8 is preferentially accumulated in the Schurig cultivar (CAN60, **Figure 3**).

##### 3.1.2.2 Cinnamic acid amides and lignanamides

A total of six cinnamic acid amides and 23 lignanamides were identified in all hemp seed accessions based on the HRMS and MS^2^ spectral match with information from the literature and online databases. N-*trans*-caffeoyltyramine (compound 11) and N-*trans*-feruloyltyramine (compound 12) represent the compounds accumulated in the highest proportion in hemp seeds out of all the detected polar metabolites. These two compounds are accumulated in the highest proportions in the French accession CAN37 and the Spanish accession Kongo Hanf (CAN58), although CAN37 accumulates higher amounts of N-*trans*-caffeoyltyramine relative to N-*trans*-feruloyltyramine, while the opposite is true for CAN58. Overall, CAN37 and CAN58 show the highest proportion of cinnamic acid amides and lignanamides among all hemp seed accessions (**Figure 3**), while an Argentinian accession (CAN51) and a Turkish accession (CAN26) both showed the lowest proportions for these two chemical classes, respectively (**Figure 3**). The Italian cultivar Eletta Campana (CAN48) and most of the accessions in cluster 4 accumulate the lowest proportion of cinnamic acid amides, while intermediate levels were found in accessions in cluster 3 (CAN42 and CAN68) followed by accessions in cluster 5 (**Figure 3**).

Similar to the monomeric cinnamic acid amides, lignanamides are accumulated in higher proportions in accessions CAN37 and CAN58, followed by the Croatian and German accessions CAN42 and CAN68, respectively, in cluster 3 and those in cluster 5 (**Figure 3**). Lignanamides constitute the most diverse group of metabolites in hemp seeds, among which cannabisin B (compound 17) and cannabisin C (compound 27) represent the two most abundant metabolites of this chemical class. Compounds in this chemical class are generally found as isomeric molecules apparently distinguishable by the intensity of their diagnostic MS^2^ ions (**Table 2**), although no study has been performed to validate this hypothesis. Interestingly, although accessions CAN42 and CAN68 are closely related based on their overall chemical compositions (**Figure 1C**), the lignanamides profile of these two accessions showed salient differences (**Figure 3**). While both accessions accumulate similar proportions of compounds 16 – 23, accession CAN68 has a much higher proportion of cannabisins D, E, F, G, N and O, relative to CAN42 (compounds 25 – 38, **Figure 3**). Interestingly, while most cinnamic acid amides and lignanamides seem to be preferentially accumulated in accessions in cluster 1 (CAN37 and CAN58), compounds 13 (N-*trans*-coumaroyl tyramine) and 14 (tricoumaroyl spermidine) were accumulated mostly in accessions CAN36 and CAN20, respectively, both in cluster 5.

##### 3.1.2.3 Cannabinoids and lipid-like molecules

Considering the previous reports and current regulations on the presence of cannabinoids in hemp, we performed target mass searches for the main cannabinoids previously reported in hemp seed oil and organic extracts (Citti et al., 2019; Jang et al., 2020). Trace amounts (< 0.3%) of only three cannabinoids were detected in 39 of the 52 hemp seed accessions: cannabidiol (CBD), tetrahydrocannabinol (THC) and tetrahydrocannabinolic acid (THCA). THC and CBD were detected in comparatively higher proportions in the Italian cultivar Eletta Campana (CAN48, **Figure 3**), while THCA was mainly detected in the accessions belonging to cluster 5 (especially CAN17 and CAN22), as well as in a Kompolti cultivar (CAN56, **Figure 3**).

Similarly, three unidentified lipid-like molecules (compounds 42 – 44, **Figure 3**) were also detected mainly in the Italian Eletta Campana (CAN48), although trace amounts were also found in other accessions. Manual inspection of MS data indicated that these metabolites likely correspond to oxidated fatty acids. However, their structural characterization is yet to be determined.

### 3.2 Structural characterization of new cinnamic acid glycosyl sulphates

**Figure 4:**
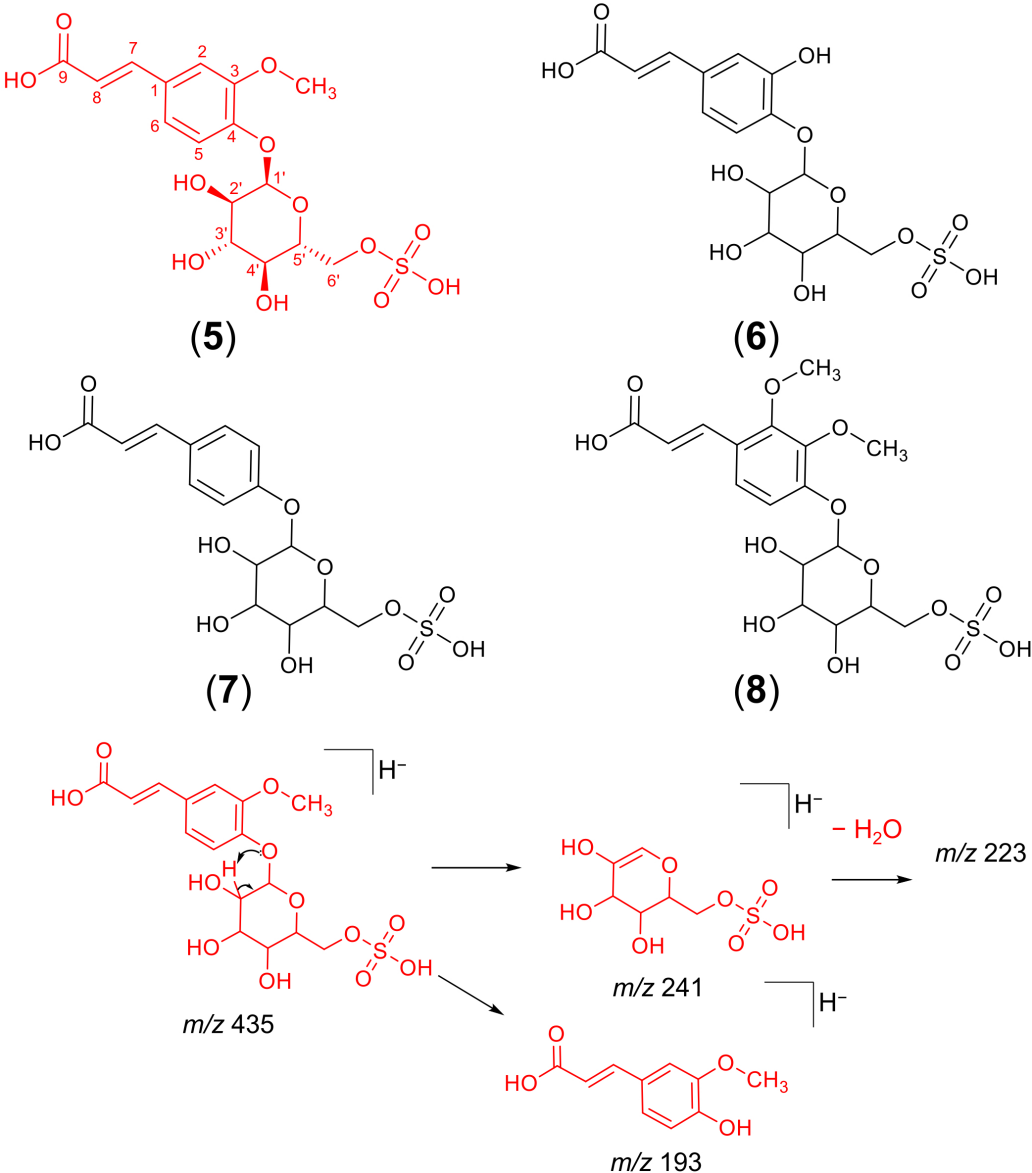
Chemical structures of cinnamic acid glycosyl sulphates detected in hemp seed and fragmentation mechanism of the isolated ferulic acid glucosyl sulphate. The chemical structure of ferulic acid-4-*O*-glucosyl-6’-*O*-sulphate (compound 5, in red) was confirmed by NMR experiments, while the chemical structures of the remaining compounds (6 – 9) were suggested by interpretation of HRMS and MS^2^ data alone and, therefore, await spectroscopic confirmation.

Considering that the analysis by FBMN and our dereplication approach suggested the presence of several potentially new metabolites belonging to a previously unreported molecular family in hemp (cinnamic acid glycosyl sulphates), we performed a target isolation of the most abundant metabolite from this chemical class, detected at 6.84 min with a mass of 435.06061 *m/z* [M−H]^−^ (**Table 2**). The process started with a dried 20% methanolic extract of hemp seeds, which was submitted to the classic isolation processes using Sephadex LH-20 column chromatography followed by semipreparative HPLC (see Materials and Methods section). Approximately 4 mg of ferulic acid 4-*O*-glucosyl-6’-*O*-sulphate (compound 5) was afforded. Furthermore, the tentative structures of four additional cinnamic acid glycosyl sulphates (compounds 6 – 9; **Figure 4**) in the crude extracts are tentatively reported based on similarity to 5 according to HRMS and MS^2^ data. All the compounds in this chemical class (compounds 5 – 9, **Figure 4**) showed a characteristic base peak ion in their MS^2^ spectra at 241 *m/z* [M−H]^−^, representing a deprotonated glucopyranosyl-sulphate unit formed after the neutral loss of the phenylpropanoid moiety. A subsequent neutral loss of a water molecule generated the ion at 223 *m/z* (**Figure 4**). In addition to these diagnostic ions, minor peaks representing a deprotonated ferulic acid (193 *m/z*), caffeic acid (179 *m/z*), cinnamic acid (163 *m/z*) and dimethoxycinnamic acid unit (223 *m/z*), were observed in the MS^2^ spectra of compounds 5, 6, 7 and 8, respectively. The suggested fragmentation mechanism leading to the formation of these diagnostic ions is provided in **Figure 4**.

Compounds 5 and 9 were assigned as two isomers of the previously undescribed ferulic acid glucosyl sulphate metabolite, based on chromatographic and spectrometric data (HRMS and MS^2^). Both compounds showed a deprotonated molecule [M−H]^−^ at *m/z* 435.0606 (**Table 2**), consistent with the molecular formula of C_16_H_20_SO_12_ (calculated for C_16_H_19_SO_12_, 435.0603). Their online UV spectra displayed absorbance maxima at 289 and 315 nm, characteristic of phenylpropanoid derivatives.

The 1H NMR spectrum of compound 5 included a proton spin system consistent with a six membered meta-para-disubstituted aromatic ring (**Table 3**), and two adjacent exocyclic olefinic monoprotonated carbons with a coupling constant (15.8 Hz) consistent with the trans configuration. The 13C spectrum is consistent with that of a substituted free ferulic acid moiety. Positions 3 and 4 of the ferulic acid moiety were assigned by HMBC couplings with other protons on the ring, and by an online NMR shifts simulator (nmrdb.org). The position of the methoxy singlet was established by a HMBC coupling to the carbon at position 3, and an NOE coupling to the proton doublet (1.2 Hz) at position 2. The attachment of the glucosyl moiety was established by a HMBC coupling from the anomeric proton to the fully substituted carbon at position 4.

**Table 3:** NMR data of ferulic acid 4-O-glucosyl-6’-O-sulphate (compound 5) in MeOD.

Feruloyl					Glucose		
No.	^13^C	1H	HMBC, H→C	No.		^13^C	1H
1	130.73	–	–	1’*		102.34	4.94, d (3.5 Hz)
2	112.37	7.22, d (1.2 Hz)	1, 3	2’		74.8	3.52, dd (3.5, 8.4 Hz)
3	150.98	–	–	3’		71.27	3.42, dd (7.6, 8.4 Hz)
OCH_3_	56.8	3.82, 3H s	3	4’		77.58	3.48, dd (7.5, 7.6 Hz)
4	149.8	–	–	5’		71.27	3.68, dd (5.9, 7.5 Hz)
5	116.3	6.69, d (7.9 Hz)	6	6’		68.15	4.13, dd (5.9, 11.1 Hz),
6	130.95	7.03, dd (1.2, 7.9 Hz)	1				4.36, d (11.1 Hz)
7	145.13	7.56, d (15.8 Hz)	2, 8, 9				
8	119.3	6.40, d (15.8 Hz)	1				
9	171.2	–	–				

*HMBC coupling observed from the proton on the anomeric 1’ to position 4 of the feruloyl moiety.

The glucosyl moiety was assigned using COESY couplings, coupling constants and HSQC. The position of attachment of the sulphate was confirmed by the re-shielding of the carbon at position 6’, which is normally measured at 61 – 62 ppm in similar glycosyl-arene structures (Schuster et al., 1986).

### 3.3 METABOLIC profiling by GC-MS

#### 3.3.1 Fatty acid profiles of different hemp seed accessions

Metabolic profiling by GC-MS of the derivatized oil extracted from 52 hemp seed accessions demonstrated homogeneous chemistry with a predominance of linoleic acid (**Table 4**). Six fatty acids were detected as the major oil components in all accessions: palmitic acid, oleic acid, linoleic acid, γ-linolenic acid, α-linolenic acid and stearidonic acid. Integration of chromatographic peak areas showed that linoleic acid (53.65% ± 2.18), α-linolenic acid (20.18% ± 3.30) and oleic acid (13.62% ± 1.86) are the three main fatty acids, representing more than 87% of the total oil components (**Table 4**). The ratio of linoleic acid and α-linolenic acid among all hemp seed accessions was found to be 2.66:1, with accessions CAN19, CAN32, CAN43, CAN44, CAN50, CAN53 and CAN54 showing the closest values to 3:1, reported as optimal for human nutrition (**Table 4**).

**Table 4:** Percentage composition of the main fatty acids found in a collection of 52 hemp seed accessions.

Accession	Palmitic acid	Oleic acid	Linoleic acid	γ-Linolenic acid	α-Linolenic acid	Stearidonic acid	LA: αLA ratio
CAN16	7.17	12.89	52.38	5.89	18.87	2.80	2.78
CAN17	7.55	11.20	53.11	4.54	21.78	1.81	2.44
CAN18	9.05	16.45	50.19	2.96	20.01	1.33	2.51
CAN19	7.49	13.54	54.27	5.27	17.57	1.86	3.09
CAN20	7.36	12.12	53.80	3.37	21.55	1.80	2.50
CAN21	7.29	13.72	53.24	2.62	21.76	1.37	2.45
CAN22	7.76	12.37	51.87	3.07	23.16	1.77	2.24
CAN23	6.11	14.20	51.69	0.00	28.00	0.00	1.85
CAN24	8.33	14.38	52.91	0.00	23.39	0.99	2.26
CAN26	8.70	12.60	54.88	0.00	22.67	1.15	2.42
CAN27	8.23	12.59	51.53	4.03	21.26	2.38	2.42
CAN28	7.70	12.55	51.22	4.04	22.83	1.66	2.24
CAN29	8.35	11.93	54.67	2.70	20.91	1.43	2.61
CAN30	7.83	17.28	55.14	4.90	13.46	1.39	4.10
CAN31	7.14	11.43	52.78	5.52	20.38	2.76	2.59
CAN32	7.99	13.44	54.79	4.07	18.23	1.48	3.01
CAN33	8.03	11.16	53.35	4.13	21.35	1.97	2.50
CAN34	6.59	12.14	58.79	4.44	16.45	1.59	3.57
CAN35	6.70	13.98	59.29	0.00	18.19	1.84	3.26
CAN36	7.30	9.46	52.02	3.05	26.28	1.91	1.98
CAN37	6.91	12.63	50.47	0.00	28.19	1.80	1.79
CAN38	7.76	14.89	54.34	4.56	16.62	1.82	3.27
CAN39	6.80	12.01	55.82	0.00	23.21	2.16	2.40
CAN40	8.60	11.98	48.84	3.93	24.48	2.16	2.00
CAN42	7.87	13.89	59.15	0.00	17.81	1.28	3.32
CAN43	6.67	12.77	54.59	5.36	18.51	2.10	2.95
CAN44	7.77	15.45	53.34	4.08	17.89	1.47	2.98
CAN45	8.07	15.14	52.80	3.43	19.33	1.23	2.73
CAN46	7.42	14.37	54.44	4.71	17.30	1.76	3.15
CAN47	8.29	15.24	51.05	2.52	21.86	1.04	2.34
CAN48	8.46	17.22	54.84	2.68	15.70	1.09	3.49
CAN50	7.29	14.13	53.24	5.79	17.82	1.72	2.99
CAN51	7.38	13.90	56.52	0.00	20.74	1.46	2.72
CAN52	7.56	15.73	52.62	3.20	19.61	1.28	2.68
CAN53	7.71	12.40	53.43	6.44	17.83	2.19	3.00
CAN54	7.84	14.01	52.65	5.48	18.02	2.00	2.92
CAN55	7.88	15.48	54.27	5.00	15.88	1.49	3.42
CAN56	6.71	15.01	55.04	0.00	21.92	1.32	2.51
CAN58	7.34	12.48	51.89	0.00	27.17	1.11	1.91
CAN59	7.80	11.06	54.49	0.00	25.12	1.53	2.17
CAN60	7.41	12.56	52.74	5.88	18.98	2.43	2.78
CAN61	8.18	19.67	53.29	3.14	14.97	0.75	3.56
CAN62	7.64	12.65	55.40	6.48	15.80	2.03	3.51
CAN63	8.35	14.80	53.31	3.22	19.19	1.12	2.78
CAN64	9.00	14.63	51.62	3.05	20.07	1.64	2.57
CAN65	7.90	13.88	55.35	5.22	15.89	1.77	3.48
CAN66	7.87	12.24	52.74	3.85	21.61	1.68	2.44
CAN67	8.06	10.87	50.71	5.93	21.86	2.58	2.32
CAN68	7.48	16.30	51.70	3.00	20.27	1.25	2.55
CAN69	7.75	13.97	54.08	3.48	18.97	1.75	2.85
CAN70	7.29	13.55	54.86	2.68	20.39	1.24	2.69
S1	8.70	13.80	58.08	0.00	18.19	1.22	3.19
Mean	7.70 ± 0.62	13.62 ± 1.86	53.65 ± 2.18	3.23 ± 2.06	20.18 ± 3.30	1.63 ± 0.51	2.66

LA, Linoleic acid; αLA, α-Linolenic acid.

**Figure 5:**
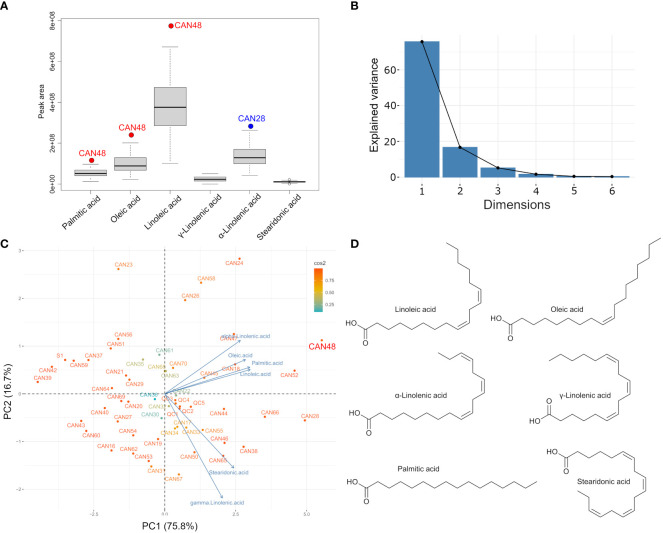
Fatty acid profile and clustering of the 52 hemp seed accessions based on their fatty acid compositions. **(A)** Boxplot showing the mean accumulation major fatty acids detected in all hemp seed accessions; **(B)** Scree plot and percentage of explained variance by the first 6 components; **(C)** PCA biplot showing the fatty acids-based clustering of all hemp seed accessions and **(D)** Chemical structures of the main fatty acids detected in hemp seeds.

Chromatographic peak area comparisons revealed that the Italian cultivar Eletta Campana (CAN48) represents the accession with the highest accumulation of fatty acids, followed by samples of the cultivar “Fibrida” (CAN52) and “Forose” (CAN28). However, these cultivars showed interesting differences in the accumulation of specific metabolites. CAN48 showed the highest concentration of palmitic acid, oleic acid and linoleic acid, while CAN28 showed the highest concentration of γ-linolenic acid, α-linolenic acid and stearidonic acid. A boxplot analysis based on the fatty acids composition further confirmed that CAN48 and CAN28 are clear outliers in the accumulation of specific lipophiles and the mean values for the six major fatty acids (**Figure 5A**). Further analysis of the same dataset by PCA revealed that the fatty acid composition of the 52 hemp seed accessions is rather homogeneous with only two PCs accounting for more than 92% of the total variance (**Figures 5B, C**). The chemical structures of the main fatty acids detected in hemp seeds are reported in **Figures 5D**.

## 4 Discussion

Hemp is an industrial crop that has been widely investigated due to its diversity of applications in the pharmaceutical, nutraceutical, food, cosmetic, textile, and material industries (Schluttenhofer and Yuan, 2017; Farinon et al., 2020). However, the chemical diversity of the hemp seed metabolome has not been explored in detail and new metabolites with unknown properties are likely to be discovered. In the current study, following an untargeted metabolomics approach by LC-MS and GC-MS, we discovered a previously unreported molecular family in hemp seeds “cinnamic acid glycosyl sulphates” and linked the presence of biologically important molecules to certain accessions. Chemical knowledge of these cultivars and accessions could be used as a base in future to optimize commercial cultivars in the context of nutritional and other functional uses.

Metabolic fingerprinting by LC-MS and data mining tools revealed that the 52 hemp seed accessions can be categorized into five main groups based on the similarities of their metabolic fingerprints. Although it is expected that when growing the same seeds under homogeneous conditions there will be some differences in the metabolic grouping, we believe that we can only have an accurate representation of the true metabolic phenotype of an organism by studying the chemical composition of plants/organs obtained from individuals collected in their natural environment. Germplasm and herbarium collections provide valuable samples for this. In the current study, among all the studied accessions and cultivars, the Spanish accession Kongo Hanf (CAN58) and the French accession CAN37 showed a remarkable structural diversity of bioactive and novel metabolites, some of them accumulated in high amounts. These two varieties showed the highest accumulation of phenylpropionamides (including cinnamic acid amides and their oxidative coupling products lignanamides), as well as new cinnamic acid glycosyl sulphates.

Phenylpropionamides (cinnamic acid amides and lignanamides) represents the main chemical class of bioactive metabolites in hemp seeds (Moccia et al., 2020; Leonard et al., 2021a; Leonard et al., 2021b). These compounds display potent antioxidant, anti-inflammatory, cytotoxic and acetylcholinesterase inhibitory activities, both *in vitro* and *in vivo* studies (Yan et al., 2015; Farinon et al., 2020; Moccia et al., 2020; Leonard et al., 2021a). For instance, previous studies have recognized the strong potential of hemp seed phenylpropionamides as protective agents against human chronic diseases (Leonard et al., 2021a), although results from clinical trials or cohort studies are not available to support this. Our results indicate that N-*trans*-caffeoyltyramine (compound 11) and N-*trans*-feruloyltyramine (compound 12) constitute the main phenylpropionamides accumulated in hemp seeds, especially in the Spanish accession Kongo Hanf (CAN58) and the French accession CAN37. These two compounds have demonstrated strong antioxidant activities (Chen et al., 2012; Moccia et al., 2020) and *in vivo* anti-neuroinflammatory action (Maiolo et al., 2018; Zhou et al., 2018). Furthermore, the lignanamides fraction of hemp seeds are also highly active. Like compounds 11 and 12, lignanamides (compounds 16 – 38) are also accumulated in high amounts in the accession CAN58 and CAN37.

Recent studies by Chen et al. (2012) observed stronger DPPH scavenging activity by cannabisin B (compound 17) isolated from hemp seed than common soy (*Glycine max* (L.) Merr.) isoflavones and secoisolariciresinol diglucoside from flax seed (*Linum usitatissimum* L.). Cannabisins M, A, C, D, and 3,3’-demethylgrossamide (compounds 21, 23, 27, 32 and 20, respectively) from hemp seed also displayed potent antioxidant activities *in vitro* (Yan et al., 2015). These compounds are especially accumulated in accessions CAN37, CAN58, CAN68 and CAN42.

The anti-inflammatory properties that these and other hemp seed lignanamides display have also been widely recognized. For example, grossamide, (compound 35) accumulated in higher amounts in accession CAN37 (**Figure 3**), showed a significant reduction in the production of the proinflammatory cytokines interleukin-6 (IL-6) and tumor necrosis factor-a (TNF-a) at a concentration of 10 – 20 µM in a lipopolysaccharide (LPS) induced model (Luo et al., 2017). These cytokines have been linked to the stimulation of inflammatory and auto-immune processes in many diseases such as rheumatoid arthritis, multiple sclerosis, psoriasis as well as different types of cancer (Bradley, 2008; Blauvelt, 2017; Shang et al., 2017). Like grossamide, cannabisin F (compound 34) has also been reported to reduce the production of IL-6 and TNF-a in LPS-induced models, although through a different mechanism (Wang et al., 2019).

Regarding the cytotoxic effects of hemp seed lignanamides, previous studies have reported that it varies in accordance with their chemical structures. For example, cannabisin B has been shown to induce autophagic cell death on HepG2, a human liver cancer cell line (Chen et al., 2013), while cannabisin F is especially active against SH-SY5Y, a human neuroblastoma cell line (Li et al., 2019). Nevertheless, other hemp seed lignanamides, such as grossamide, cannabisin D and cannabisin G, have displayed weak cytotoxic activity against human prostate cancer LNCaP cells (Ma et al., 2002). Considering that CAN37, CAN58, CAN68 and CAN42 have a higher concentration of bioactive cinnamic acid amides and lignanamides, these accessions could be commercially prioritized as potential sources of antioxidant, anti-inflammatory and cytotoxic metabolites or in selective breeding programs to improve the concentration of specific metabolites in other commercialized cultivars. Interestingly, none of the five historic cultivars with deleted status in the EU database (**Table 1**) showed a particularly high concentration of bioactive metabolites compared to the newer accessions.

In addition to the 29 phenylpropionamide peaks annotated in the current study (**Table 2**), our results suggest that other lignanamides might also be present. Manual inspection of the FBMN results indicate that there are at least eleven additional nodes representing potentially new phenylpropionamides, some of them showing *m/z* values previously unreported in hemp seeds. For example, two nodes at *m/z* 600.270 and *m/z* 614.286 [M+H]^+^ appear to be structurally related to tricoumaroyl spermidine (compound 14). Manual interpretation of MS data suggests that the node at *m/z* 600.270 [M+H]^+^ likely represents a hydroxylated analogue, while the node at *m/z* 614.286 [M+H]^+^ is likely its methoxylated version. Several isomers of *m/z* 597.33 and *m/z* 611.23 [M+H]^+^ were also observed. The isolation and complete structural characterization of these potentially new metabolites is still necessary. Recent studies have proved that despite being exhaustively commercialized, hemp seed continues to be a promising source of new lignanamides. For example, Yan et al. (2015) identified the first trimeric feruloyltyramine in hempseeds, of which its coupling resulted in the formation of an 8-*O*-4 linkage. Considering that hemp hulls are the main site of accumulation of lignanamides, future agricultural waste valorisation programs are highly encouraged. Hemp hulls are a by-product of the hemp seed oil industry (Pojić et al., 2014; Mattila et al., 2018), which represents an opportunity. This is further supported by the fact that the total lignanamides in hemp hull can be up to four times higher than in whole hemp seed (3.36 mg/g vs 0.77 mg/g, respectively) (Mattila et al., 2018).

Aside from phenylpropionamides, hemp seed oil is also a commodity held in high regard for its nutraceutical properties due to a perfectly balanced content of omega 3 and omega 6 polyunsaturated fatty acids (Crescente et al., 2018; Citti et al., 2019). Mean concentration values for the six main fatty acids detected in the current study: palmitic acid oleic acid, linoleic acid, γ-linolenic acid, α-linolenic acid and stearidonic acid (**Table 4**), are within ranges comparable to previous studies (Galasso et al., 2016; Pavlovic et al., 2019). The Italian cultivar Eletta Campana (CAN48) showed the highest accumulation of fatty acids among the investigated hemp seed accessions. However, CAN32, CAN50 and CAN53 were the accessions with the ratio of linoleic acid: α-linolenic acid closest to 3:1, reported as optimal for human nutrition (Leizer et al., 2000; Simopoulos, 2008; Galasso et al., 2016). These results agree and further expand the studies of Galasso et al. (2016). Similar to previous studies, the cultivar with the lowest proportion of α-linolenic acid was Eletta Campana (CAN48) (Galasso et al., 2016). However, while the mean concentration values for the six fatty acids are also within comparable ranges to previous reports (Galasso et al., 2016; Pavlovic et al., 2019), there are some discrepancies in the concentration of specific fatty acids in certain accessions. For example, we found a higher proportion of oleic acid in CAN48 than in CAN39 and the higher percentage of α-linolenic acid was found in CAN40 instead of CAN24 as reported by Galasso et al. (2016). These discrepancies can be due to different extraction methods and the fact that Galasso et al. (2016) studied the oil composition of seeds coming from individuals grown under the same conditions while our study directly analysed the seeds stored in the IPK genebank.

In addition to the main fatty acids previously mentioned, other fatty acids have also been reported in minor concentrations in hemp seed oil (Galasso et al., 2016; Pavlovic et al., 2019). These metabolites were not reported in the current paper due to identification and quantification discrepancies in these low concentrated metabolites. However, considering that the biological properties of hemp seed oil are related to the presence of the major fatty acids, especially the ratio between linoleic acid and α-linolenic acid, our results are biologically relevant. Complete reports of minor metabolites in hemp seed oil can be found in other studies (Galasso et al., 2016; Pavlovic et al., 2019).

A recent study reported the cannabinoid profile of ten commercially available hemp seed oils (Citti et al., 2019). Besides tetrahydrocannabinol and cannabidiol, another 30 cannabinoids were also identified (Citti et al., 2019). In the current study, we found traces of only three cannabinoids using a non-selective LC-MS method. Our results demonstrated that Eletta Campana (CAN48) was the cultivar with the highest concentration of cannabinoids, although their concentration was around 200 times lower than that of the most abundant metabolite (N-*trans*-caffeoyltyramine). Previous studies on the same accession (CAN48) obtained from the same genebank (IPK) reported an absolute quantification value of THC of 0.08%, which is well below the 0.3% threshold set by international regulations (Citti et al., 2019). Based on the low THC content of this cultivar, we might assume that all the hemp seed accessions included in the current study meet the international regulatory standards of < 0.3% THC content.

Recent studies have proved that despite being heavily studied, hemp seed continues to be a promising source of new metabolites. In the current study we discovered a previously unknown molecular family in hemp seeds “cinnamic acid glycosyl sulphates” widely distributed across all hemp seed accessions in similar concentrations. The identity of one novel compound from this chemical class was confirmed by isolation and interpretation of spectroscopic data, and four structural analogues, three of them new, is also suggested. The presence of these compounds in all hemp accessions suggest that cinnamic acid glycosyl sulphates might have important implications as quality control markers for the authentication of commercial hemp seed products extracted with highly aqueous solvents. Considering that these highly polar extracts, commonly used in the manufacture of cosmetics, are usually devoid of characteristic hemp seed phenylpropionamides due to insolubility issues, the need of more suitable quality control markers is justified. Further studies are still needed to identify the biological properties of these new metabolites.

In conclusion, our results confirm that some hemp accessions present in the IPK germplasm collection appear to contain more interesting profiles of metabolites than some of the cultivated hemp cultivars, as first suggested by Galasso et al. (2016). We suggest that accessions CAN37 and CAN58, should be scientifically prioritized for the discovery of new metabolites as well as in selective breeding programs for the development of new cultivars with high contents of bioactive phenylpropionamides. However, despite the functional and nutraceutical properties of this crop, the seeds of hemp are not totally free of antinutritional compounds, such as phytic acid (Russo, 2013; Galasso et al., 2016). According to previous reports, including ten of the accessions investigated here, CAN48 presents the highest levels of phytate, although other cultivars not included here like Futura75 and Felina32 have even higher values. The presence of high phytate contents in some cultivars limit their use in human nutrition and other monogastric animals since a high level of phytic acid may lead to mineral deficiencies of macro-and microelements, protein digestibility and poor organoleptic properties (Russo, 2013; Russo and Reggiani, 2015; Galasso et al., 2016). Therefore, an improvement for this trait might be necessary if the high yielding oil cultivar CAN48 is prioritized. According to previous studies (Galasso et al., 2016), the Italian accession CAN40 might be a good candidate to reduce the content of phytate through hybridization and selective breeding. Other accessions such as CAN32, or the Fibrimon cultivars CAN50 and CAN53, could also be prioritized if a 3:1 ratio of linoleic acid/α-linolenic acid is desired.

## Data availability statement

The datasets presented in this study can be found in online repositories. The names of the repository/repositories and accession number(s) can be found below: https://massive.ucsd.edu/ProteoSAFe/static/massive.jsp, MSV000090725.

## Author contributions

GP-G conceived and designed the study, performed the LC-MS and GC-MS data analysis, guided the isolation methodology and wrote the first draft of the manuscript. AR prepared the samples for LC-MS and GC-MS acquisition and assisted in the isolation of metabolites. NS elucidated the structure of the new metabolite and assisted in the writing and proofreading of the manuscript. MC assisted in the extraction and isolation of the new metabolite and MS provided financial assistance for LC-MS and GC-MS analyses and assisted in the writing and proofreading of the manuscript. All authors contributed to the article and approved the submitted version.
