# Supplementary material for: Mining the chemical diversity of the hemp seed (Cannabis sativa L.) metabolome: discovery of a new molecular family widely distributed across hemp
Source: Front Plant Sci. 2023 Aug 10;14:1114398. doi: 10.3389/fpls.2023.1114398 (PMC10449600; doi:10.3389/fpls.2023.1114398)
Supplement: Supplementary file 1 [file DataSheet_1.docx]

Supplementary Material

Mining the chemical diversity of the hemp seed (Cannabis sativa L.) metabolome: discovery of a new molecular family widely distributed across hemp

Guillermo Federico Padilla-González^1*^, Abigail Rosselli^1^, Nicholas J. Sadgrove^1,2^, Max Cui^1^, Monique S.J. Simmonds^1^

^1^Royal Botanic Gardens, Kew, London, United Kingdom.

^2^Department of Botany and Plant Biotechnology, University of Johannesburg (Auckland Park Campus), Auckland Park, 2006, Johannesburg, South Africa.


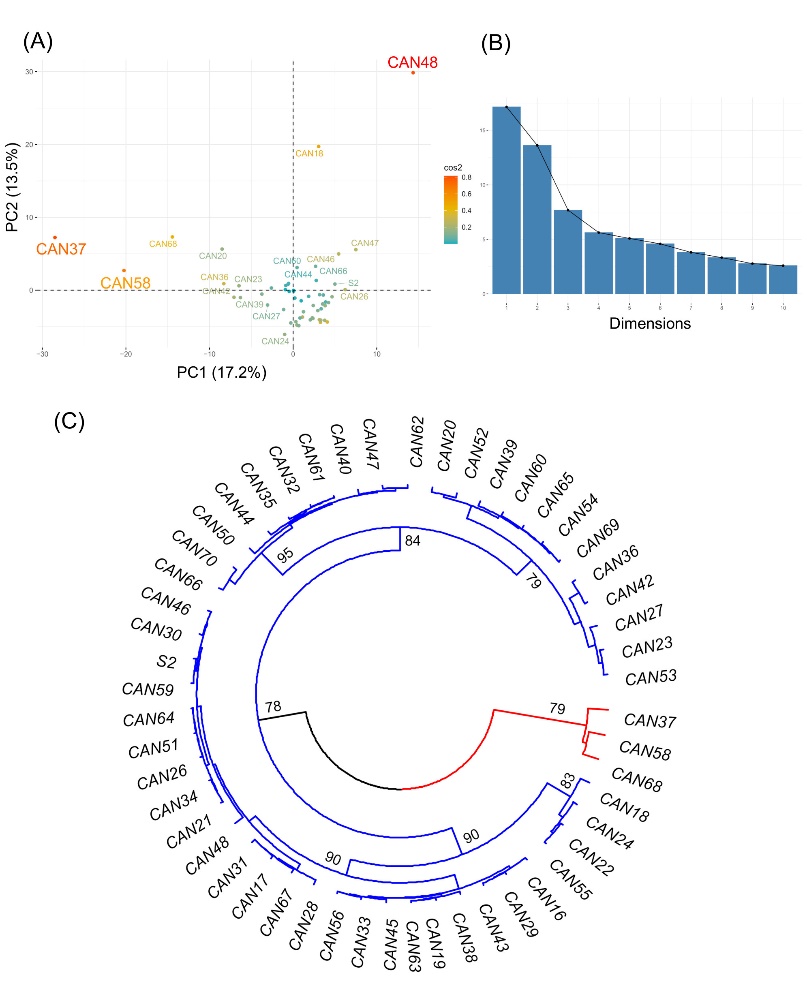


**Supplementary Figure 1. Clustering of the fifty-two hemp seed accessions based on the similarities of their metabolic fingerprints detected by LC-MS in negative ion mode**. (A) PCA scores plot showing accessions CAN48, CAN58 and CAN37 as outliers; (B) Scree plot and the percentage of variance explained by the first 10 components; (C) HCAbp showing the metabolic clustering of the 52 hemp seed accessions.


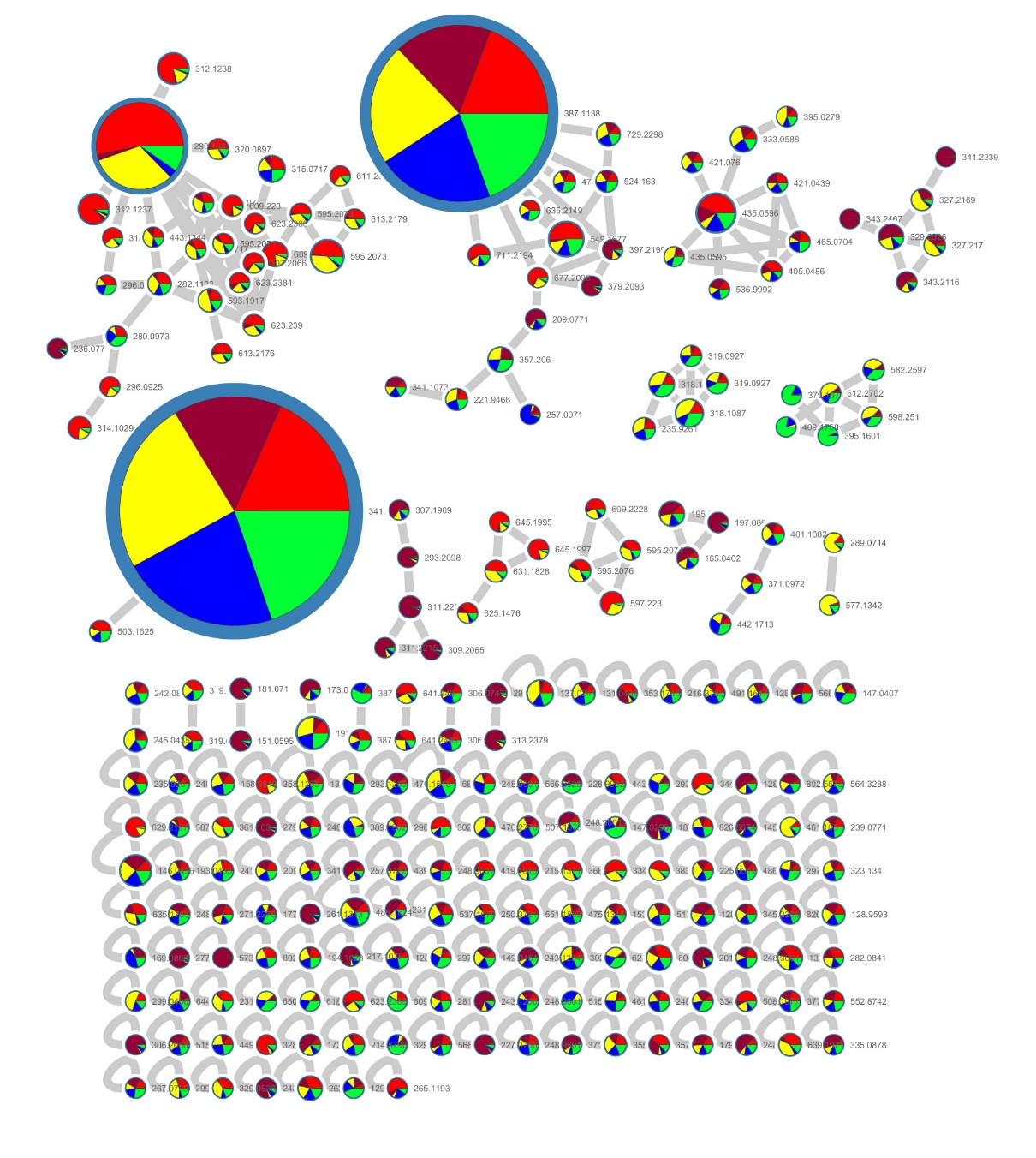


**Supplementary Figure 2. Feature Based Molecular Networking (FBMN) of LC-MS data in the negative ionization mode.**
